# Design of coherent wideband radiation process in a Nd^3+^-doped high entropy glass system

**DOI:** 10.1038/s41377-022-00848-y

**Published:** 2022-06-14

**Authors:** Linde Zhang, Jingyuan Zhang, Xiang Wang, Meng Tao, Gangtao Dai, Jing Wu, Zhangwang Miao, Shifei Han, Haijuan Yu, Xuechun Lin

**Affiliations:** 1 Laboratory of All-solid-state Light Sources, Beijing Engineering Research Center, Institute of Semiconductors, Chinese Academy of Sciences, 100083 Beijing, China; 2 Synlumin Conuninex (Shanghai) Enterprise Development Co., Ltd., 201401 Shanghai, China; 3 Time-wave-space Optical Technology (Xiaogan) Co., Ltd., 432012 Xiaogan, Hubei China; 4 High-dimensional Plasma Sources Technology (Xiaogan) Co., Ltd., 432012 Xiaogan, Hubei China

**Keywords:** Optical physics, Optical physics

## Abstract

We discover that the spatially coherent radiation within a certain frequency range can be obtained without a common nonlinear optical process. Conventionally, the emission spectra were obtained by de-exciting excited centers from real excited energy levels to the ground state. Our findings are achieved by deploying a high-entropy glass system (HEGS) doped with neodymium ions. The HEGS exhibits a much broader infrared absorption than common glass systems, which can be attributed to be high-frequency optical branch phonons or allowable multi-phonon processes caused by phonon broadening in the system. A broadened phonon-assisted wideband radiation (BPAWR) is induced if the pump laser is absorbed by the system. The subsequent low-threshold self-absorption coherence modulation (SACM) can be controlled by changing excitation wavelengths, sample size, and doping concentrations. The SACM can be red-shifted through the emission of phonons of the excited species and be blue-shifted by absorbing phonons before they are de-excited. There is a time delay up to 1.66 ns between the pump pulse and the BPAWR when measured after traveling through a 35 mm long sample, which is much longer than the Raman process. The BPAWR-SACM can amplify the centered non-absorption band with a gain up to 26.02 dB. These results reveal that the shift of the novel radiation is determined by the frequency of the non-absorption band near the absorption region, and therefore the emission shifts can be modulated by changing the absorption spectrum. When used in fiber lasers, the BPAWR-SACM process may help to achieve tunability.

## Abstract (web summary)

Designable spatially coherent frequency conversion by synergistic effect of phonon broadening and self-absorption modes in high-entropy glass system.

## Graphical abstract

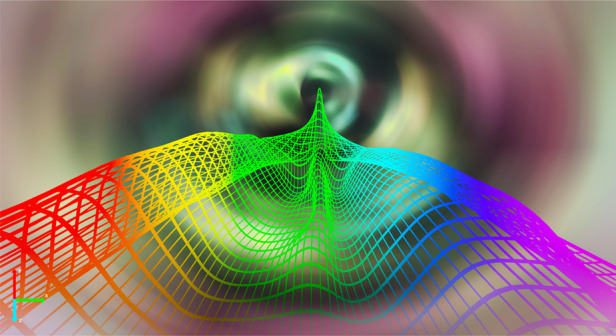

## Introduction

The vibrational modes of the solid matrix are often directly involved in the de-excitation process of the excited center, i.e., phonon-assisted radiation if depicted by a quasi-particle picture^1^. When the phonon energy of the matrix is high, and the Huang-Rhys factor between the matrix and the excited center is large, the typical non-radiative transition process, such as the multi-phonon emission, can even dominate the de-excitation process^2,3^. Even if the phonon energy of the matrix is relatively low, the excited center can de-excite to one excited state with a lower energy level and then emit fluorescent photons through radiation^4^. When the excitation energy is sufficiently high, the excited centers can directly absorb or emit phonons at virtual energy levels and then eventually de-excite to the ground state through the radiation process. This is a typical Raman process and can directly reflect the vibrational modes of the solid matrix^5^. The resonance Raman results from linear electron-phonon coupling and the equilibrium geometry changes along the phonon mode upon excitation^6,7^. In the radiative de-excitation process of excitons, the phonons can change the band structure locally due to the deformation of the atomic lattice (deformation-potential scattering). It is so-called phonon side-band emission^8^. In addition, some dark excitons cannot directly recombine due to momentum-forbidden. They can only be indirectly recombined by absorbing or emitting phonons and emit observable signals^9^. The discovery of these complex radiation processes proves the importance of phonon-assisted radiation.

For crystalline materials with long-range orders^10^, the phonon modes involved in the phonon-assisted radiation process generally involve one or several phonons with specific vibration frequencies^1,3,11,12^. Phonons in one material can be altered due to phonon-phonon interactions^13^ and chemical disorder^14^. In some glassy materials, the phonon modes broaden in a short range^15–17^. However, the locally distinct chemical environments or mass disorder in high-entropy systems can induce an anharmonic phonon-phonon coupling and composition disorder, which leads to significant phonon broadening^14,18^. So far, phonon scattering was mainly used to explore high-entropy materials’ thermal and transport characteristics^18–20^. The terminology of high-entropy comes from the high configuration entropy larger than 1.5 R (R is the universal gas constant), which results from randomly distributed multiple nearly equal components in a crystal lattice^21^. The earliest high-entropy system is high-entropy alloys (HEAs) comprising at least five principal metal elements in equal or near-equiatomic ratios^21^. This reference presented the first confirmed case of an entropy-driven transition to a homogeneous, single-phase system. It also boosted a new family of high-entropy materials (HEMs), i.e., high-entropy metallic glasses^22^, high-entropy ceramics^23^, and high-entropy composites^24^, etc. The design concept of HEMs provides extensive tunability through composition and processing technology, which endows the material with desirable mechanical and thermal properties. So far, phonon broadening has been mainly used to explore the thermal and transport characteristics of HEMs^18–20^. Since many HEMs have no transparency in the visible light range, research on the phonon-assisted radiation of HEAs is rather limited. Then several questions arise: Is the design strategy of HEAs applicable to transparent glass systems to yield a high-entropy glass system (HEGS)? If yes, how is the phonon broadening in the HEGS? How do the broadened phonons interact with the excited center? What are the dynamics of phonon-assisted radiation in HEGS?

With these questions in mind, we applied the high-entropy strategy in an alternative glass phase system to realize a class of Nd^3+^ ions doped high-entropy glass system (HEGS) and intentionally designed its emission spectra by controlling absorption spectra. The HEGS exhibits a complex structure with tetrahedral voids filled by different ions, including Li^+^, Zn^2+^, Si^4+^, P^5+^, S^6+^, etc. In this way, the tetrahedral voids of the system exhibit highly random interstitial features, resulting in extremely high configuration entropy. The absorption spectrum of the HEGS indicates the presence of high-frequency optical phonons or allowable multi-phonon processes in the system, which makes it exhibit a much stronger and wider infrared absorption than conventional glass system. A novel radiative de-excitation process occurs in the HEGS, and the emission spectrum correlates well with the absorption spectrum. Unlike conventional fluorescence, Raman scattering, or frequency multiplication process, this process shows spatial coherence at a low excitation threshold. The time delay characteristics observed in the HEGS in this work differ from those in conventional radiation processes. Considering the phonon broadening feature in the absorption spectrum, we identify that the radiative de-excitation process consists of a broadened-phonon-assisted wideband radiation (BPAWR) process and a subsequent self-absorption coherence modulation (SACM) process, i.e., BPAWR-SACM. We modulated the BPAWR-SACM process by varying the excitation wavelength, the sample size, and the doping concentration. Pumping the Nd^3+^ ions to different excited states with different lasers, we find that the area ratio between the red-shifted and blue-shifted emission peaks depends on the energy level density surrounding the excited state levels. We demonstrated a new kind of signal amplification through the novel BPAWR-SACM process based on these findings. In the conventional stimulated Raman^25,26^, a combination of specific wavelengths is needed for signal amplification. Conversely, dual- or even multi-wavelength amplification of any non-absorption band of HEGS is achievable. When the pump lasers are injected into the system, if the system exhibits certain absorption near a non-absorption band, the energy from the pump lasers can be transferred to the non-absorption band for signal amplification. It can be expected that the BPAWR-SACM process helps to achieve tunability of optical fiber laser and supercontinuum. With a continuous-wave (CW) laser as pump, a modulated emission with wide-spectrum and spatial coherence can be obtained by designing the absorption spectrum of the system. Therefore, one can easily obtain CW white light lasers by utilizing the BPAWR-SACM process. The repetition frequency and pulse width of white light can be tuned by a Q-switched or mode-locked way, converting the white light lasers to short pulse light for more applications.

## Results

### Design strategy of HEGS and its phonon broadening characteristics

A high-entropy system’s design strategy is to randomly distribute multiple nearly equal components in the crystal lattice, resulting in a significant increase in the configuration entropy of the system, even greater than the melting entropy of the system itself^27^. Under such circumstances, the proportions of each atom in the composite system are similar, and they all tend to be evenly distributed in space, forming a single-phase system^28^. The corresponding unit cell can easily form a simple stacked body-centered cubic (bcc) unit cell when the atom size differs. Otherwise, it is easy to form a relatively dense face-centered cubic (fcc) unit cell^29^.

In this study, the HEGS was designed based on the close packing of oxides. From a mesoscopic view, many tetrahedral and octahedral voids are formed through close packing of O^2-^. Tetrahedral voids can be occupied by two classes of ions. According to Pauling’s first rule, one type of ions should have simple geometric structures. The radius ratio between anions and cations is 0.225-0.414, and the corresponding interstitial ions (i.e., Si^4+^, P^5+^, S^6+^) have approximately radii of 31.5 pm to 58.0 pm. Other ions (i.e., Li^+^, Zn^2+^) have a strong ability to induce polarization, which can cause lattice deformation for occupying interstices. Cations with a large radius such as K^+^ and Nd^3+^ can occupy the corresponding octahedral voids for the charge balance in a unit cell. A schematic drawing of the glass structure is presented in supplementary Fig. S1.

**Fig. 1:**
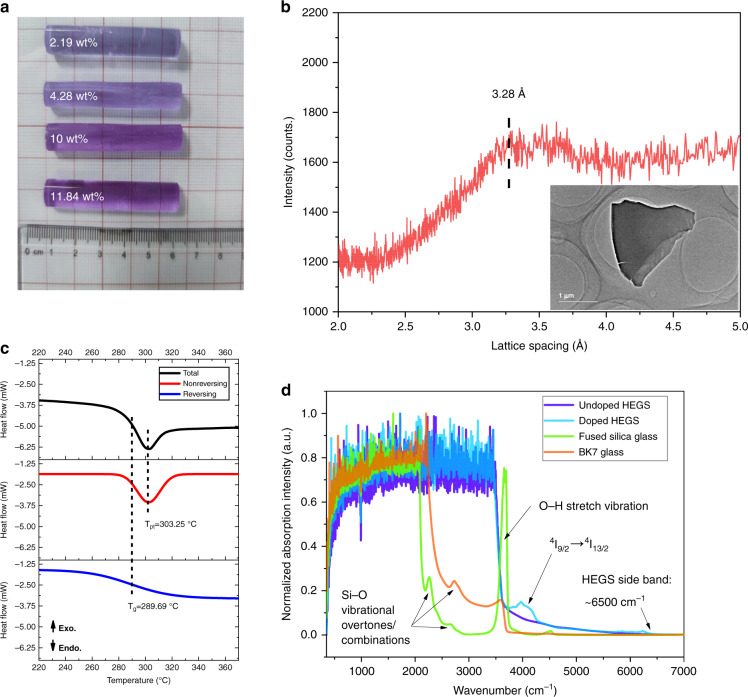
Structure, thermodynamics, and spectral properties of HEGS. **a** Photo of HEGS samples with varying doping concentrations. **b** The intensity-interplanar relationship of the HEGS system, derived from the original intensity-diffraction angle relationship from X-ray diffraction measurement. The inset is the TEM micrograph of the HEGS sample. **c** DSC result of one HEGS sample. When the sample was heated to around 300 °C, a non-reversing endothermic peak was present, accompanying the reversing vitrification process. Isolation of the two processes was accomplished by peak fitting. **d** The infrared absorption of different samples with a thickness of 3-4 mm. For fused glass, the peak at 2200 cm^−1^ results from the double-frequency peak of Si-O-Si vibration mode at 1104 cm^−1^. The peak at 2670 cm^−1^ results from the double-frequency peak of Si-O-Si vibration mode at 795 cm^−1^ and the sum-frequency peak of Si-O-Si vibration mode at 1104 cm^−1^. The peak at 3654 cm^−1^ is assigned to the O-H due to the adsorption of water on the fused silica. Overtones with higher frequency is unlikely to appear and the allowable overtones or combinations are limited. Hence, the absorption of fused silica glass is weak between 2000 cm^−1^ and 3800 cm^−1^

Figure 1a shows the digital image of the HEGS rods with varying doping concentrations of NdCl_3_·6H_2_O. The intensity-interplanar relation (Fig. 1b) proves that the HEGS has a weakly ordered structure. The inset inside shows no particulates precipitation in the HEGS, and the overall contrast remains consistent. An endothermic peak in the differential scanning calorimetry (DSC) result (Fig. 1c) can be explained by enthalpy relaxation^30,31^. Near the vitrification temperature, the relatively ordered structure in the HEGS disintegrates, and the corresponding free volume expands, which causes enthalpy relaxation and a non-reversing endothermic peak. This also corresponds to a non-convergent order-disorder phase transition^32^. With KBr pellet method, the infrared absorption of undoped HEGS differs little from that of HEGS (Supplementary Fig. S2). Compared with fused silica, the FWHM at 468 cm^−1^ and 795 cm^−1^ of the HEGS is broader, and the peaks can be assigned to Si-O-Si vibration modes^33^. This results from the presence of many vibration modes of oxygenated groups (i.e., Zn-O, Si-O-Si, Li-O, P-O-P, etc.) with comparable frequencies in the HEGS. The overlapping frequencies of their local vibration modes greatly affect the corresponding overtones and combinations^34^. That is to say, the normal mode of collective vibration in the HEGS will widen its frequency range, leading to the broadening of phonon modes. This can be supported by the result in Fig. 1d. The absorption frequencies of fused silica are characterized by: a descending peak starting at 2000 cm^−1^, weak peaks near 2200 cm^−1^ and 2600 cm^−1^, an extremely strong peak around 3654 cm^−1^ and a side band peak ending at 3800 cm^−1^. The infrared absorption in the region of 2250-3700 cm^−1^ of BK7 glass is stronger than silica glass, but much weaker than HEGS. This is because the added composition like B and As in BK7 glass richens its vibration modes, and hence permits the Si-O vibrational overtones/combinations. Nevertheless, the allowable phonon modes in BK7 glass are less than that in the HEGS which consists of more different components. The major difference between the undoped HEGS and doped HEGS lies in a weak absorption at 3970 cm^−1^, which can be assigned to Nd^3+^ transition from ^4^I_9/2_ to ^4^I_13/2_ (Fig. 1d, Supplementary Fig. S3). Both the undoped HEGS and HEGS exhibit a strong absorption in the region of 400-3600 cm^−1^, certain absorption in the region of 3600-6500 cm^−1^, and a side band ending at 6500 cm^−1^. The infrared absorption side-band is much higher than other glass systems^15–17^. This can be attributed to the complicated couple relation between the vibration modes of oxygenated groups in the HEGS, which broadens and strengthens multiple-frequency and sum-frequency overtones. The high-frequency optical phonons and allowed multi-phonon process are present in the HEGS due to phonon broadening. Finally, the absorption band of the HEGS was broadened, showing a strong absorption in the mid- and near- infrared range. Phonon broadening in the HEGS also leads to optical phonon modes with frequencies much higher than that in conventional solid matrixes^35,36^. We expect that these high-frequency optical phonons or allowable multi-phonon processes will participate in the corresponding optical processes and have a significant impact on radiation process.

### Broadened phonon-assisted wideband radiation (BPAWR) and self-absorption coherence modulation (SACM)

**Fig. 2:**
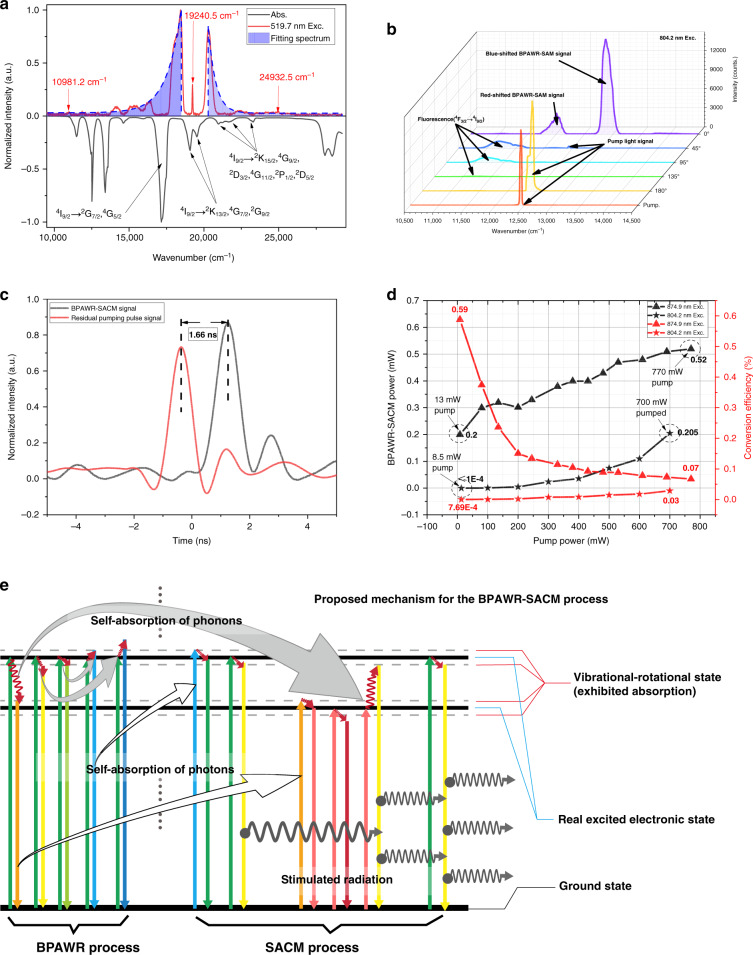
Photo-physical properties of BPAWR-SACM process. **a** Normalized emission spectrum (upper part) and normalized absorption spectrum (lower part) in the visible light range. The corresponding transition modes of Nd^3+^ ions are marked. The fitting spectrum represents the hypothesized emission spectrum with non-absorption. **b** Emission spectra at different measurement angles relative to the forward direction of the excitation light. The corresponding excited energy levels are marked in the figure. **c** Time-delay measurement between the residual pump pulse and the generated frequency-shifted radiation. **d** BPAWR-SACM signal power and conversion efficiency versus pump power of laser excitation at 874.9 nm (denoted as 874.9 nm Exc.) and 804.2 nm (denoted as 804.2 nm Exc.). **e** The suggested mechanism for the BPAWR-SACM process

Experimentally, we measured the normalized absorption spectrum and the normalized emission spectrum in the forward direction in the visible range using a green diode laser at 519.7 nm as the excitation source. The results are presented in Fig. 2a. The high-frequency optical phonon modes or allowable multi-phonon process play an important role in the phonon-assisted radiation process in the visible range. Apart from residual excitation light signals, the emission spectrum (upper part in Fig. 2a) also includes some new frequency components that show significant red-shift or blue-shift from the excitation frequency. These new frequency components have no relation with the fluorescence of Nd^3+^ ions since they cannot be attributed to the emission spectrum of Nd^3+^ transition between any two energy levels^37,38^. Moreover, the red-shift and blue-shift frequency ranges are about 10000 cm^−1^ and about 7000 cm^−1^, respectively. The asymmetric shift is also different from the conventional Raman shift^39,40^. It can also be found that there is a certain correlation between the emission spectrum and the absorption spectrum (lower part in Fig. 2a). The peaks indicated by black arrows represent the excitation process of Nd^3+^ ions’ transition from the ground state to the excited state. Extremely low emission intensity is detected, corresponding to these absorption peaks.

In contrast, the peaks indicated by red arrows correspond to either no absorption or weak absorption in the absorption spectrum. It is supposed that the newly generated red-shifted and blue-shifted components compose quasi-continuous wideband emission peaks, which corresponds to a BPAWR due to energy transfer of Nd^3+^ ions in the other excited states. However, due to the self-absorption of some frequency components by Nd^3+^ ions, the wideband emission peaks are fragmented into a series of different frequency components, exhibiting discontinuous and asymmetric features. Therefore, the newly generated frequency components result from the combined effect of BPAWR and the subsequent self-absorption modulation (SAM).

We also measured the emission spectra at different angles relative to the forward direction of the excitation lasers for spatial coherence study (Fig. 2b, Supplementary Fig. S4). The results show that the red-shifted and blue-shifted BPAWR-SAM signal can only be observed when the measurement is conducted in the forward direction. At the measurement angle turns 45° from the forward direction, a weak scattered excitation signal and an emission peak at 11199 cm^−1^ are present. This emission peak can be attributed to the corresponding fluorescence emission of Nd^3+^ ions de-excited from ^4^F_3/2_ to ^4^I_9/2_. In detail, the Nd^3+^ ions are excited to energy level ^4^F_5/2_ from ^4^I_9/2_, de-excited to energy level ^4^F_3/2_ by phonon emission, and return to ground state ^4^I_9/2_ by emitting light. At the measurement angles of 135° and 180°, the fluorescence emission peak intensity from ^4^F_3/2_ to ^4^I_9/2_ is extremely weak. These results prove that the BPAWR-SAM process has strong spatial coherence. Since the BPAWR process alone has no spatial coherence, the observed spatial coherence should be attributed to SAM. Therefore, the SAM process can be named self-absorption coherence modulation (SACM), and the overall process is referred to as BPAWR-SACM.

A time delay study between the excitation laser and the generated radiation using picosecond mode-locked Ti: Sapphire laser proved that the BPAWR process differs from the conventional radiation process (Fig. 2c). The corresponding optical experiment setup and obtained BPAWR-SACM spectra were presented (Supplementary Figs. S5 and S6). With part of the light pulse from the pump laser as the trigger of the oscilloscope, the time delay between the residual pump pulse and the BPAWR-SACM signal is measured to be 1.66 ns after the pump laser passing through a 35 mm sample. While for the conventional Raman and stimulated Raman process, the time delay generally ranges from 100 fs to ~ 1 ps, rarely up to 10 ps^41–44^. The longer time delay indicates that the novel radiation is not a Raman scattering.

We also studied the conversion efficiency of the BPAWR-SACM process by measuring the ratio between the output power and the pump laser power (Fig. 2d), and the emission spectra were also acquired (Supplementary Fig. S7). With a pump laser of 8.5 mW at 874.9 nm, the corresponding BPAWR-SACM power was relatively weak (only 0.05 mW centered at 852.7 nm), but the conversion efficiency was as high as 0.58%. By increasing pump laser to 770 mW, the BPAWR-SACM power increased and reached 0.52 mW, but the conversion efficiency decreased dramatically to 0.07%. On the other hand, with a pump power of 13 mW at 804.2 nm, the corresponding BPAWR-SACM power was lower than the instrument’s detection limit. It increased to 0.205 mW when the pump power was increased to 700 mW. The corresponding conversion efficiency increased with increasing laser power, and the maximum value was 0.03%. The results show that the BPAWR-SACM process has an extremely low threshold, and laser power in the order of ten milliwatts is enough to excite the process. It also indicates that the emission is not a common nonlinear process since the conventional nonlinear process needs extremely high light intensity for signal generation^45^. The varied conversion efficiency at different excitation wavelengths could be attributed to the different absorption intensities. The low output power and the peculiar behaviors of conversion efficiency can be attributed to the thermal effect in the mean free path of phonon modes involved in the de-excitation of BPAWR-SACM. At room temperature, the mean free path of the phonons is relatively short, and phonons are consumed into heat by remarkable phonon scattering. This fast reduction of phonons causes fewer phonons to contribute to the subsequent radiation process. It is expected that the conversion efficiency will be improved if the HEGS is cooled down.

We proposed a mechanism for the peculiar BPAWR-SACM process to facilitate the interpretation of the process (Fig. 2e). When an excitation laser excites the excited centers in the HEGS medium to one excited state with a real energy level, it greatly enhances the phonon-electron interaction between the excited centers and the medium. Many excited centers can either be de-excited by emitting phonons and successively emitting red-shifted photons with various frequencies or absorbing phonons and emitting blue-shifted photons. Since the high-frequency phonon modes or allowable multi-phonon process can cover a broad absorption spectrum up to thousands of wavenumbers, the corresponding emission spectrum of the phonon-assisted radiation process also shows broadening by thousands of wavenumbers. Such a process is the so-called BPAWR as described above, which contains several absorption spectra between different electronic and surrounding vibration-rotation excited states. When the BPAWR process occurs in the excited centers, the ground state adjacent to the excited centers can directly absorb the phonons emitted at a specific wavelength and transit to the corresponding excited state, leading to the photons’ self-absorption process. The excited state returns to the ground state by a multi-phonon-assisted process or emitting a photon at a shifted frequency with phonons’ assistance. Regardless of the de-excitation route, the photons’ self-absorption process will lead to a loss of the BPAWR emission until it exhibits a discontinuous spectrum. Meanwhile, the phonons produced by the de-excitation process can be absorbed by other excited states, facilitating them to transit to other higher excited states, i.e., the phonons’ self-absorption process. The wideband radiation is permitted to propagate only in the non-absorption band of the medium, which consists of the blue-shifted band produced by the phonons’ self-absorption and the red-shifted band produced by the phonons emission. Both of them cause stimulated radiation from other excited states when propagating within the medium, thus amplifying the signal intensity of the residual band in the BPAWR process and imparting coherent properties to the corresponding band. This process is so-called SACM. The frequency components are changed accompanying the radiation propagation in the SACM process, which causes mode competition in the radiation process^46^. The SACM process induced by selecting absorption modes causes the phonon modes involved in the BPAWR process to depend on the surrounding density of allowable excited states. That is why the BPAWR and SACM processes are inseparable.

### Modulating the BPAWR-SACM process based on absorption characteristics

**Fig. 3:**
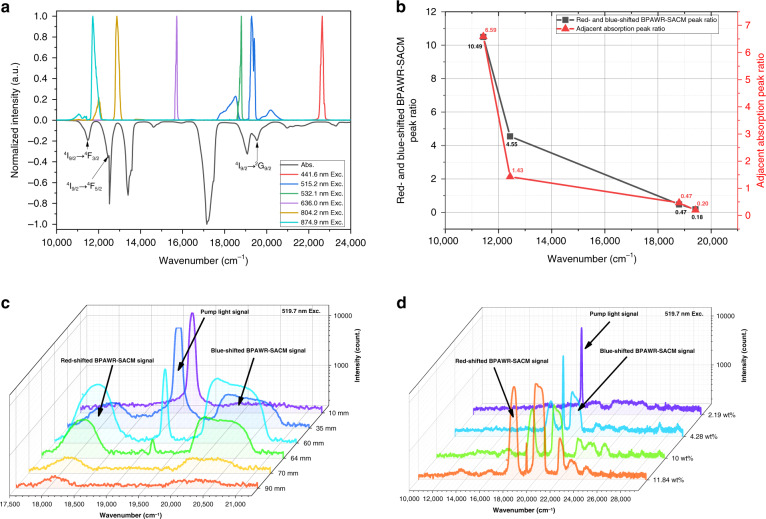
Modulation results of BPAWR-SACM process based on absorption characteristics. **a** Normalized emission spectra at different excitation wavelengths (upper part) and normalized absorption spectrum in the visible light range (lower part). Corresponding excited energy levels are marked in the figure. **b** Statistical results of the red-shifted and blue-shifted BPAWR-SACM peak area ratio versus the adjacent absorption peak area ratio. All relevant excited states within phonon frequency of 3000 cm^−1^ were considered in the statistics. **c** Emission spectra were measured by 519.7 nm excitation at samples with different lengths. **d** Emission spectra of samples with different doping concentrations excited by 519.7 nm laser. The intensities in **c**, **d** were presented in logarithm to observe relatively weak emission peaks

The proposed mechanism of BPAWR-SACM in Fig. 2e suggests that the excitation wavelengths that can induce the BPAWR-SACM process correspond to the strong absorption in the absorption spectrum of Nd^3+^ ions transition in each energy level. A weak absorption in the absorption spectrum cannot induce the BPAWR-SACM process, even BPAWR alone. We also studied the influence of excitation wavelengths in the BPAWR-SACM process by measuring the emission spectra (upper part in Fig. 3a) and extracting the spectral shift of the BPAWR-SACM signals relative to the excitation light (Supplementary Fig. S8). When the sample is excited by lasers at 441.6 nm, 532.1 nm, and 636.0 nm, respectively, only the residual pump lights are observed. In contrast, the residual pump lights are relatively weak or unobservable when pumping with other excitation wavelengths such as lasers at 515.2 nm, 804.2 nm, and 874.9 nm. Still, the red-shifted or blue-shifted signals can be produced. The lasers at the wavelengths mentioned above can excite Nd^3+^ from ^4^I_9/2_ in the ground state to ^2^G_9/2_, ^4^F_5/2_, and ^4^F_3/2_ in the excited state, respectively. Further statistics to the data in Fig. 3a show that the area ratio of red-shifted to blue-shifted BPAWR-SACM signal at different excitation wavelengths is positively correlated with the absorption peak area-ratio between adjacent higher excited state and lower excited state (Fig. 3b). The above results can be attributed to the surrounding energy level density determining the area and intensity of one emission peak, as suggested in Fig. 2e. These results support that by inducing BPAWR-SACM with lasers at different wavelengths, tuning radiation frequency within thousands of wavenumbers is possible.

Beer-Lambert law describes that the absorption intensity is proportional to the optical path length and the doping concentration of the medium. It inspires us to tune the SACM by changing the sample’s length, and the corresponding emission spectra are shown in Fig. 3c. For a 10 mm sample, the red-shifted or blue-shifted BPAWR-SACM signals are not observed, but only a strong signal of the residual excitation light. When the glass length is extended to 60 mm, a red-shift at 770 cm^−1^ and a blue-shift at 1074 cm^−1^ can be observed. Now the intensity of red-shifted or blue-shifted BPAWR-SACM signals reaches the maximum and starts to decrease if the sample becomes longer. For a 70 mm sample, the excitation signal disappears, and the BPAWR-SACM signals continue attenuating. The results suggest that when an excitation laser enters the HEGS medium, it is absorbed and attenuated continuously during propagation. Such an attenuation process accompanies the generation of the BPAWR-SACM before the excitation is completely consumed. At the same time, the newly generated red-shifted and blue-shifted signals continue to propagate forward until the BPAWR-SACM signals leave the HEGS or are completely absorbed by the medium. The optical path difference between the excitation laser and the BPAWR-SACM signal is around 60 mm. The light attenuation process supports that the BPAWR-SACM process in the HEGS is not formed instantaneously but is accumulated and gradually enhanced during the propagation. Therefore, the intensity ratio of emission peaks can be regulated by changing the optical path’s length.

The effects of the doping concentration on the BPAWR-SACM process were also investigated (Fig. 3d). When the doping concentration is 2.19 wt%, the excitation light intensity is strong, while the blue-shifted BPAWR-SACM signal is too weak to be distinguished from the signal noise. The strongest red-shifted BPAWR-SACM signal is located at 2666 cm^−1^, much weaker than the excitation light. With increasing doping concentration, the intensity of BPAWR-SACM signals is enhanced. Some relatively weak red-shifted or blue-shifted BPAWR-SACM peaks can also be observed in the emission spectra. When the doping concentration reaches 11.84 wt%, the intensities of both the red-shifted and the blue-shifted BPAWR-SACM signals are greatly enhanced and become much higher than that of the residual excitation light. The above data support that increasing doping concentration enhances the absorption of the pump light of the system.

### Signal amplification of centered non-absorption band due to dual-wavelength pump

**Fig. 4:**
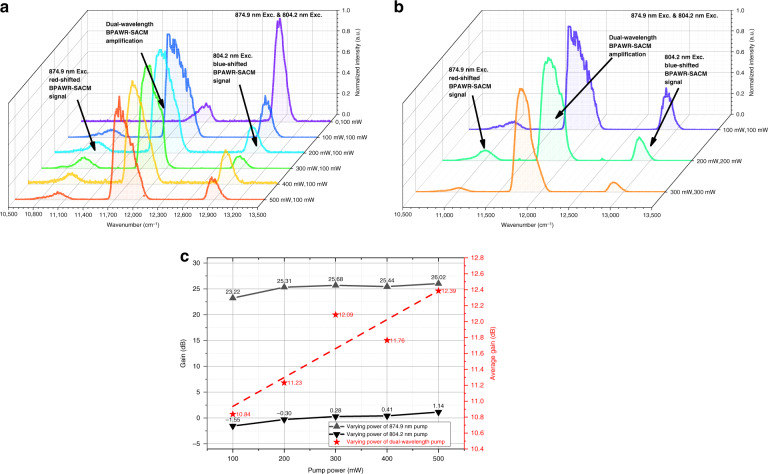
Signal amplification by the dual-wavelength pump. **a** Normalized emission spectra were obtained by maintaining the laser power at 804.2 nm constant and varying the laser’s power at 874.9 nm alone. **b** Normalized emission spectra were obtained by keeping the dual-wavelength pump power at the same level. **c** The signal gain versus pump power. The gain was calculated from the amplification produced by keeping one laser power constant while increasing another alone. Average gain was calculated from the amplification produced by synchronously increasing the dual-wavelength pump power

In the conventional stimulated Raman^26^, a combination of specific wavelengths is needed for signal amplification. As long as certain absorption occurs near a non-absorption band, the energy of the dual- or even multi-wavelength pump lasers can be transferred to the non-absorption spectrum for signal amplification^47^. This work demonstrated a new kind of signal amplification in the centered non-absorption band by a dual-wavelength pump. The experiments were done by keeping one laser’s power at 100 mW and varying the power of another laser alone (Fig. 4a, Supplementary Fig. S9) or controlling the dual-wavelength pump power at the same level (Fig. 4b). The red-shifted and blue-shifted wavenumbers in Fig. 4a, b are listed in Supplementary Table 1. As shown in Fig. 4a, when the 874.9 nm laser is off, the red-shifted BPAWR-SACM signal excited at 804.2 nm starts at 12130.2 cm^−1^ (i.e., 824.4 nm), reaches a peak at 12004.9 cm^−1^, and ends at 11701.1 cm^−1^. This signal, viewed as the target signal, is significantly weaker than the blue-shifted BPAWR-SACM signal. With a pump power of 500 mW of 874.9 nm laser, the peak of the target signal is found at 11757.9 cm^−1^, showing a red-shift. Correspondingly, the target signal ends at 11589.3 cm^−1^ (i.e., 862.9 nm). Compared with the laser at 804.2 nm, the laser at 874.9 nm effectively amplifies the target signal.

As shown in Fig. 4b, at a power of 100 mW, the target signal starts at 12130.2 cm^−1^, reaches a peak at 11696.9 cm^−1^, and ends at 11589.3 cm^−1^. As the dual-wavelength pump power is increased simultaneously, the target signal region remains unchanged, but the peak position is slightly blue-shifted. When the power reaches 300 mW, the peak is found at 11757.9 cm^−1^. Taking the red-shifted component from 874.9 nm excitation and the blue-shifted component from 804.2 nm excitation of the BPAWR-SACM as a reference, one can find that the target signal region can be significantly enhanced. These results suggest that when two lasers having similar frequencies are incident on the HEGS sample, most energies are transferred to the centered non-absorption band and significantly amplify the signal in the range of 824.4 nm to 862.9 nm. For the dual-wavelength pump with the excitation at 874.9 nm, the blue-shifted BPAWR-SCAM signal between 824.4 nm and 862.9 nm can serve as a seed light^48^, and it greatly amplifies the red-shifted BPAWR-SACM signal by the pump at 804.2 nm. As a result, the signal amplification occurred at the centered non-absorption band, which included amplification from the red-shifted BPAWR-SACM signal created by the pump at 804.2 nm and the blue-shifted BPAWR-SACM signal created by the excitation at 874.9 nm. Synchronously increasing the pump powers of the two wavelengths can also minimize the displacement of the central peak position, thereby avoiding inconsistent gain effects.

Based on the data from BPAWR-SACM signals (Fig. 4a, b, Supplementary Fig. S9), the gain of one laser relative to another laser and the average gain of the dual-wavelength pump were calculated (Fig. 4c). When the red-shifted 804.2 nm Exc BPAWR-SACM signal served as a target signal, the gain from the amplification of the blue-shifted 874.9 nm Exc BPAWR-SACM signal increased with the pump power, and the maximum gain can be as high as 26.02 dB at a pump power of 500 mW. A similar trend was also found when the 874.9 nm Exc blue-shifted BPAWR-SACM signal served as a target signal. But the gain from the amplification of the 804.2 nm Exc red-shifted BPAWR-SACM signal only had a maximum value of 1.14 dB. When the dual-wavelength pump power was increased synchronously, overall, the average gain increased with the total pump power, and the maximum gain reached 12.39 dB. The dual-wavelength pump can significantly amplify the centered non-absorption band between the dual wavelengths. The amplification efficiency of the blue-shifted BPAWR-SACM signal is higher than that of the red-shifted BPAWR-SACM signal. Such a phenomenon is mainly attributed to the fact that the absorption of the 804.2 nm excitation is much stronger than the excitation at 874.9 nm. The corresponding conversion efficiency at 804.2 nm excitation is also lower (Fig. 2d). Resultantly, the gain is higher in the case of 874.9 nm excitation.

## Discussion

This work demonstrates a novel spatially coherent wideband radiation process, which can be designed by the absorption spectra of the HEGS without using a resonator. After pump laser is absorbed by the material, the BPAWR process occurs due to high-frequency optical phonons or allowable multi-phonon processes in the system. Subsequently, stimulated emission occurs in the non-absorption band while the emission corresponding to the absorption band causes mode competition as loss, finally leading to a strong spatially coherent radiation. This subsequent SACM process is influenced by changing excitation wavelengths, sample size, and doping concentrations. Overall, the BPAWR-SACM process differs from conventional nonlinear optical process in the frequency shifts, time delay between the pump laser and emission, the intensity ratio of red- and blue-shifted components, as well as its low threshold. Pumping the Nd^3+^ ions to different excited state levels with different lasers, we find that the area ratio between the blue-shifted and red-shifted emission peaks depends on the energy level density surrounding the excited state levels. Multiple pump lights with different frequencies can amplify signal beam, and therefore the frequency requirement for pump beams will be much lower. This poses a major advantage over Raman amplification, which can only be achieved by using pumps with a specific wavelength or a combination of some wavelengths. Since the process can generate blue-shifted emission with a relatively high gain without changing nonlinear frequency, it is expected to obtain unusual visible or ultraviolet lasers by an infrared laser pump if large-size and high-quality samples can be obtained. Furthermore, energy transfer and amplification of the ultrashort pulse can also be carried out by BPAWR-SACM. The energy of single pulse can be boosted by combining chirped pulse amplification (CPA) technology. With the BPAWR-SACM process, frequency modulation in the visible light range is possible, thereby realizing the wavelength division multiplexing communication technology. Therefore, the BPAWR-SACM is expected to be applied in supercontinuum, laser amplification, and optical communication, etc.

## Materials and methods

### Preparation of Nd^3+^ doped HEGS

The recipe of the HEGS is (32.97)Zn(H_2_PO_4_)_2_·2H_2_O-(22.2)P_2_O_5_-(11.55)TEOS-(19.32)K_2_SO_4_-(X)NdCl_3_·6H_2_O-(2.66)LiOH·H_2_O-(1.23)CaCl_2_ (wt%) where X represents a variable value. The mass fractions of neodymium chloride with different amounts are 2.19 wt%, 4.28 wt%, 10 wt%, and 11.84 wt%, respectively. The mixture was prepared in Al_2_O_3_ crucible and melted at 700 °C to a flowing state and then poured into a mold. The corresponding glass samples were obtained by annealing, which was implemented: firstly, lower the sample temperature from 700 °C to 300 °C at a cooling rate of 20 °C/min. Holding for a time, it then lowers the temperature from 300 °C to 250 °C at a cooling rate of 10 °C/min. When the sample’s temperature is uniform, cool the sample to 200 °C at the rate of 10 °C/min. Finally, the sample is cooled down to room temperature at a rate of 1 °C/min. The whole process was performed in an air atmosphere.

### Characterization techniques

X-ray diffraction (XRD) patterns of HEGS samples were characterized by a powder diffractometer (X’Pert3 Powder) at a scanning rate of 5.7°/min and a step size of 0.026° (Co K_*α*_ radiation, λ = 0.179 nm). Differential scanning calorimetry (DSC, SDT Q600) was carried out by heating 50 mg of glass powder in a nitrogen atmosphere at a rate of 10 °C/min. Microstructure characterization was performed by transmission electron microscopy (TEM, FEI Tecnai G2 F30) with an accelerating voltage of 300 kV.

### FTIR measurement

The infrared absorption of doped HEGS, undoped HEGS, fused silica glass and BK7 glass (GWH21-038) was measured by an FTIR spectrometer (PerkinElmer, Spectrum Two). The experiments were conducted in two ways. One way is to grind the samples into powder and measure their respective infrared absorption with standard KBr pellet method. Another way is to directly measure the infrared absorption of different samples whose thickness is 3-4 mm. The mid-infrared to near-infrared absorption spectra of undoped HEGS and doped HEGS were obtained by a UV-VIS-NIR spectrometer (Agilent, Cary 5000).

### High-power emission spectrum measurement

The emission spectrum was measured by a fiber optic spectrometer from one end of a 65 mm HEGS sample pumped by a continuous wave (CW) semiconductor laser at 519.7 nm with a power of 1000 mW from another end. All the emission spectra were measured in this way, if not otherwise specified.

### Spatial coherence measurement

An 804.2 nm laser of power 400 mW was used to excite one HEGS sample with a length of 35 mm. The fiber optic spectrometer’s fiber port was placed on a circle of radius 20 mm, centered on the midpoint of the sample rod. By defining the side where the laser was located as 180° and the opposite side as 0°, the corresponding emission spectra were measured at an angle of 0° to 180° with an interval of 45°. Similar experiments were conducted with a 515.2 nm laser of power 400 mW and an 874.9 nm laser of power 400 mW.

### Time-delay dynamics measurement

We employed a CW 532 nm laser to pump Ti: Sapphire and generated mode-locked 802.0 nm infrared light for excitation. The mode-locked laser pulses had a pulse width of 15 ps, one single pulse energy of 3 nJ, a repetition frequency of 86 MHz, and average power of 30 mW. As shown in supplementary Fig. S5, the infrared light was split into two beams with a 45° half mirror. The reflection light, serving as a reference signal, was measured by an oscilloscope (Agilent technologies DSO7104B with bandwidth of 1 GHz and sample rate of 4 G Sa/s) following photodetector A (Thorlabs DET210 with bandwidth of 0.35 GHz). The transmission light was converted by photodetector B (Thorlabs DET210 with bandwidth of 0.35 GHz) and measured by the oscilloscope if the sample was absent. Such a signal was referred to as residual pump laser pulse. Using one HEGS sample with a length of 35 mm, we first confirmed its BPAWR-SAM characteristics with a fiber optic spectrometer and then measured the BPAWR-SAM signal by the oscilloscope.

### Threshold of BPAWR-SACM signal power measurement

A CW 874.9 nm diode laser with a power of 8.5 mW to 770 mW was coupled into a 35 mm HEGS sample. The spectral characteristics of the BPAWR-SACM process can be observed. When the excitation light was completely absorbed, an optical fiber spectrometer was replaced with a laser power meter to measure the power corresponding to the BPAWR-SACM signal. Similar experiments were conducted with an 804.2 nm laser with a power of 13 mW to 700 mW.

### Emission spectrum measurement at a single wavelength excitation with varying wavelengths

A 35 mm HEGS sample was excited by lasers with wavelengths varying from 441.6 nm to 874.9 nm with a constant power of 400 mW. The corresponding emission spectra were measured with a fiber optic spectrometer.

### Emission spectrum measurement at a single wavelength excitation with varying sample sizes

Using a 519.7 nm semiconductor laser with a power of 1000 mW as the excitation, we tested the HEGS samples with lengths ranging from 10 mm to 90 mm. The corresponding emission spectra were measured with a fiber optic spectrometer.

### Emission spectrum measurement at a single wavelength excitation with varying doping concentration

We employed a 1000 mW diode laser at 519.7 nm to excite 65 mm HEGS samples doped with Nd^3+^ ions. The doping concentration ranged from 2.19 wt% to 11.84 wt%. The corresponding emission spectra were measured with a fiber optic spectrometer.

### Emission spectrum and signal power measurement at dual-wavelength excitation with varying excitation power

A half mirror, CW diode lasers of 874.9 nm and 804.2 nm were employed to pump a 35 mm HEGS sample. The transmitted combined beam was used to generate the BPAWR-SACM process, and the emission spectra were measured with a fiber optic spectrometer. Then a laser power meter replaces the fiber optic spectrometer to measure the power of the BPAWR-SACM process. The power of the incident light was monitored in real-time with a laser power meter that received the signal from the reflected combined beam. The corresponding emission spectra and signal power were analyzed in three cases. Firstly, maintaining the pump power of 874.9 nm laser at 100 mW, we increased the pump power of 804.2 nm laser from 0 mW to 500 mW. Then, the pump power of 804.2 nm laser was kept at 100 mW, the pump power of 874.9 nm laser was adjusted from 0 mW to 500 mW. Lastly, the pump powers of both the 874.9 nm laser and the 804.2 nm laser were adjusted simultaneously.

## Supplementary information

Supplementary information
