# Supplementary material for: Design of coherent wideband radiation process in a Nd3+-doped high entropy glass system
Source: Light Sci Appl. 2022 Jun 14;11:181. doi: 10.1038/s41377-022-00848-y (PMC9197846; doi:10.1038/s41377-022-00848-y)
Supplement: Supplementary file 1 — Supplementary information [file 41377_2022_848_MOESM1_ESM.pdf]

Supplementary Information for

Design of coherent wideband radiation process in a Nd^{3+}-doped high entropy glass system

Linde Zhang[1], Jingyuan Zhang[1], Xiang Wang[2], Meng Tao[3], Gangtao Dai[4], Jing Wu[3], Zhangwang Miao[1], Shifei Han[1], Haijuan Yu[1], Xuechun Lin[1*]

[1]Laboratory of All-solid-state Light Sources, Beijing Engineering Research Center, Institute of Semiconductors, Chinese Academy of Sciences; 100083 Beijing, China.

[2]Synlumin Conuninex (Shanghai) Enterprise Development Co., Ltd.; 201401 Shanghai, China.

[3]Time-wave-space Optical Technology(Xiaogan) Co., Ltd.; 432012 Xiaogan, Hubei, China.

[4]High-dimensional Plasma Sources Technology(Xiaogan) Co., Ltd.; 432012 Xiaogan, Hubei, China.

*Corresponding author. Email: xclin@semi.ac.cn

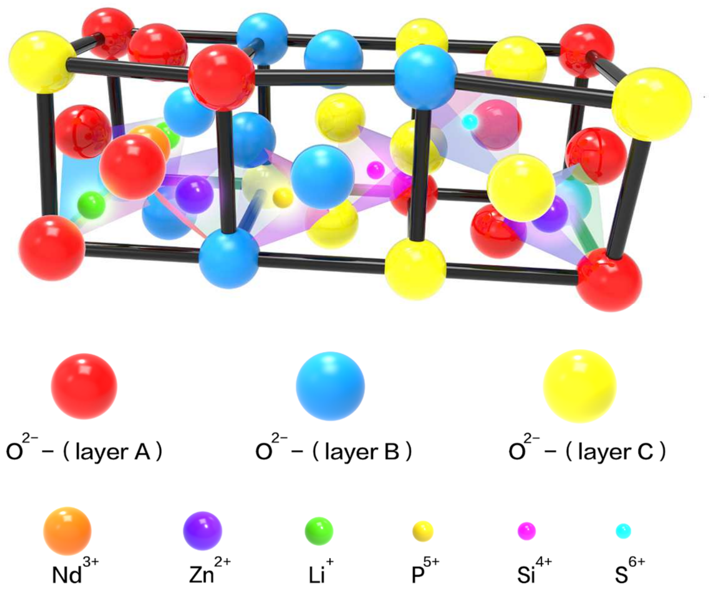

Fig. S1. A schematic drawing of the structure of the doped HEGS. Many tetrahedral and octahedral voids are formed through close packing of oxides. Tetrahedral voids can be occupied by two classes of ions. One type of interstitial ions (i.e., Si^{4+}, P^{5+}, S^{6+}) have approximately radii of 31.5 pm to 58.0 pm. Other ions (i.e., Li^{+}, Zn^{2+}) have a strong ability to induce polarization and lattice deformation. Cations with a large radius such as K^{+} and Nd^{3+} can occupy the corresponding octahedral voids for the charge balance in a unit cell.

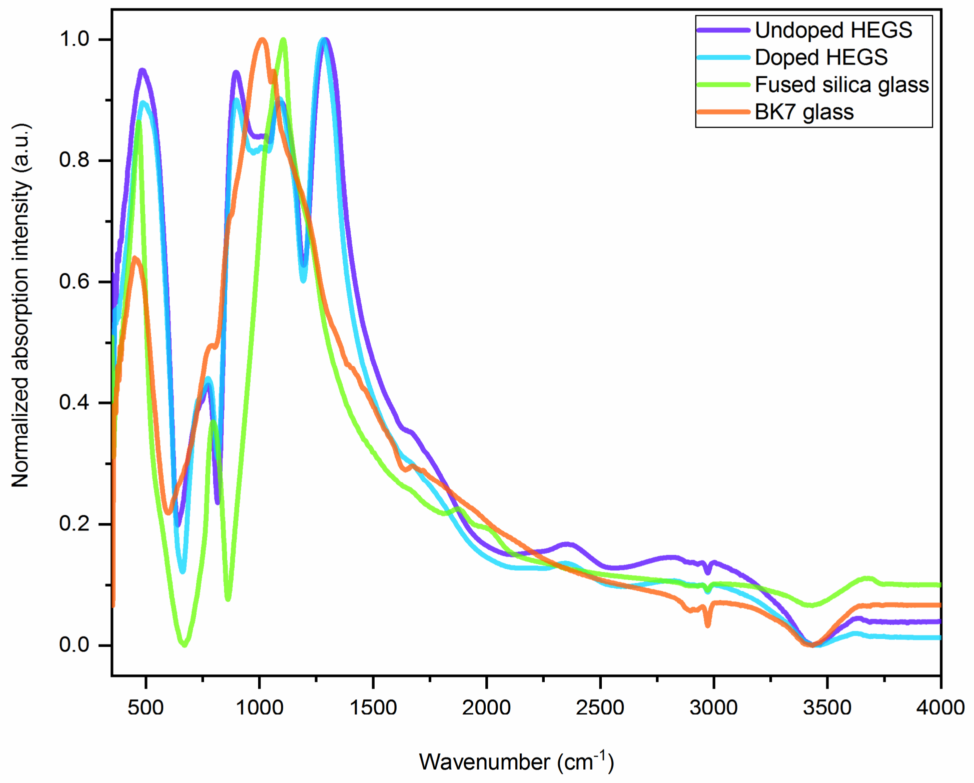

Fig. S2. The infrared absorption of different samples measured with standard KBr pellet method. Both the undoped HEGS and HEGS exhibit absorption with a wide FWHM at 483 cm^{-1} (assigned to vibration modes of Li-O, Zn-O, P-O-P and Si-O-Si), 540 cm^{-1} (assigned to vibration modes of Zn-O and Si-O-Li), 720 cm^{-1} (assigned to vibration mode of Li-O), 775 cm^{-1} (assigned to vibration modes of P-O-P and Si-O-Si), 896 cm^{-1} (assigned to vibration modes of P-O-P and Si-O-Si), 1091 cm^{-1} (assigned to vibration modes of S-O-S, P-O-P and Si-O-Si) and 1295 cm^{-1} (assigned to vibration mode of P=O). One system's phonon mode can be viewed as the normal mode of collective vibration formed by these local vibration modes in the long range. For fused silica glass, the Si-O-Si vibration mode appears at 900-1300 cm^{-1}, and the normal mode of collective vibration can be decomposed into corresponding longitudinal optical (LO) and transverse optical (TO) phonons.

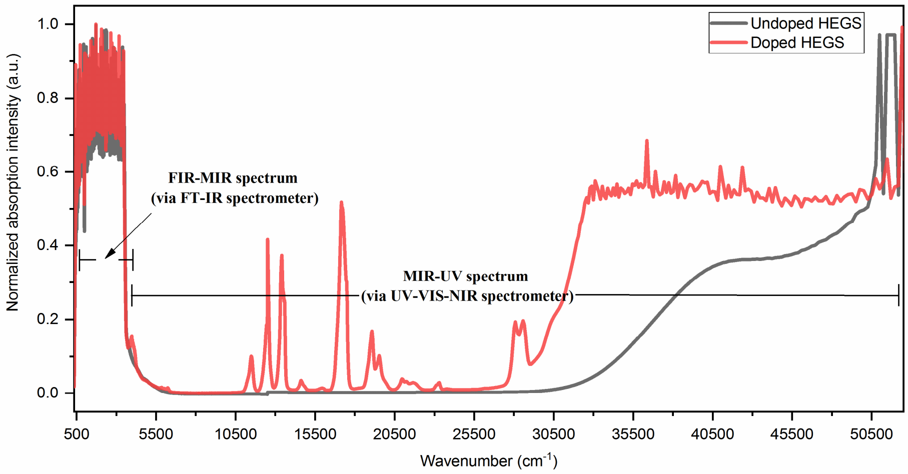

Fig. S3. The extended absorption spectrum of undoped HEGS and doped HEGS from far-infrared to near-infrared. FIR-MIR absorption spectrum was obtained by FTIR measurement, and the MIR-NIR absorption spectrum was obtained by UV-VIS-NIR measurement. The two absorption spectra were normalized and then combined to get the extended absorption spectrum. The absorption spectrum above 10000 cm^{-1} of doped HEGS represents the Nd^{3+} ions' absorption.

a

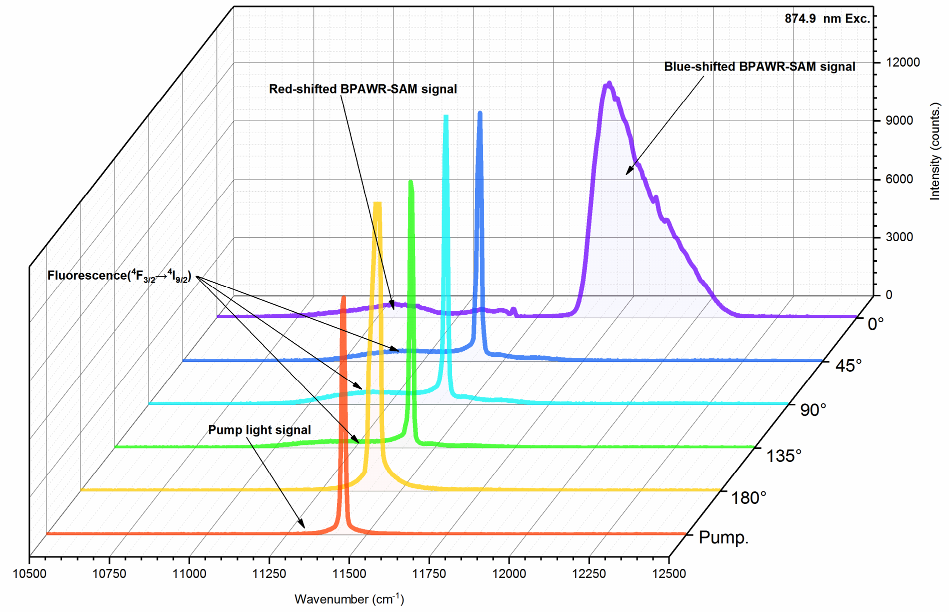

b

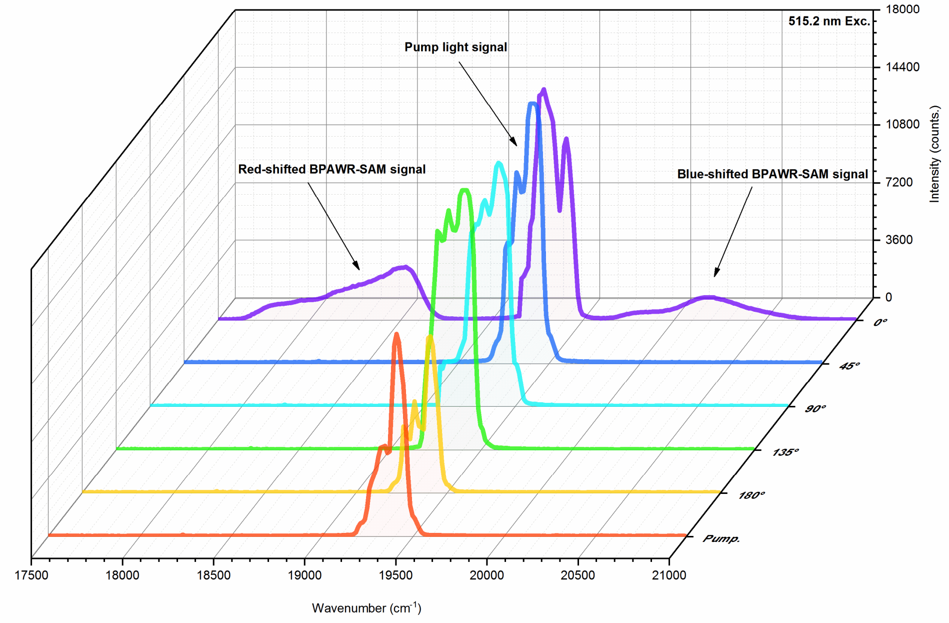

Fig. S4. Emission spectra at different measurement angles relative to the forward direction of the 874.9 nm excitation (a) and 515.2 nm excitation (b). The corresponding excited energy levels are marked in the figure.

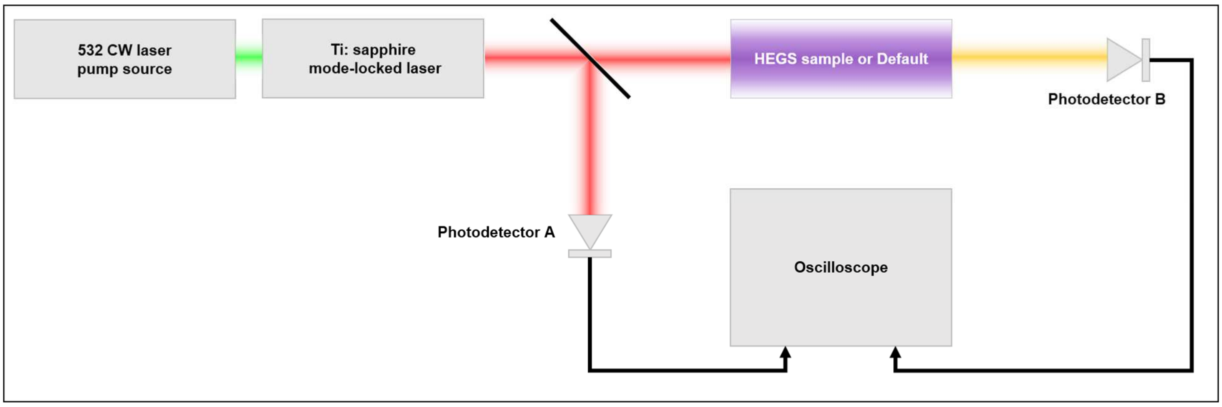

Fig. S5. Optical configuration of time-delay dynamics measurement. We employed a CW 532 nm laser to pump Ti: Sapphire and generated mode-locked 802.0 nm infrared light for excitation. The mode-locked laser pulses had a pulse width of 15 ps, one single pulse energy of 3 nJ, a repetition frequency of 86 MHz, and average power of 30 mW.

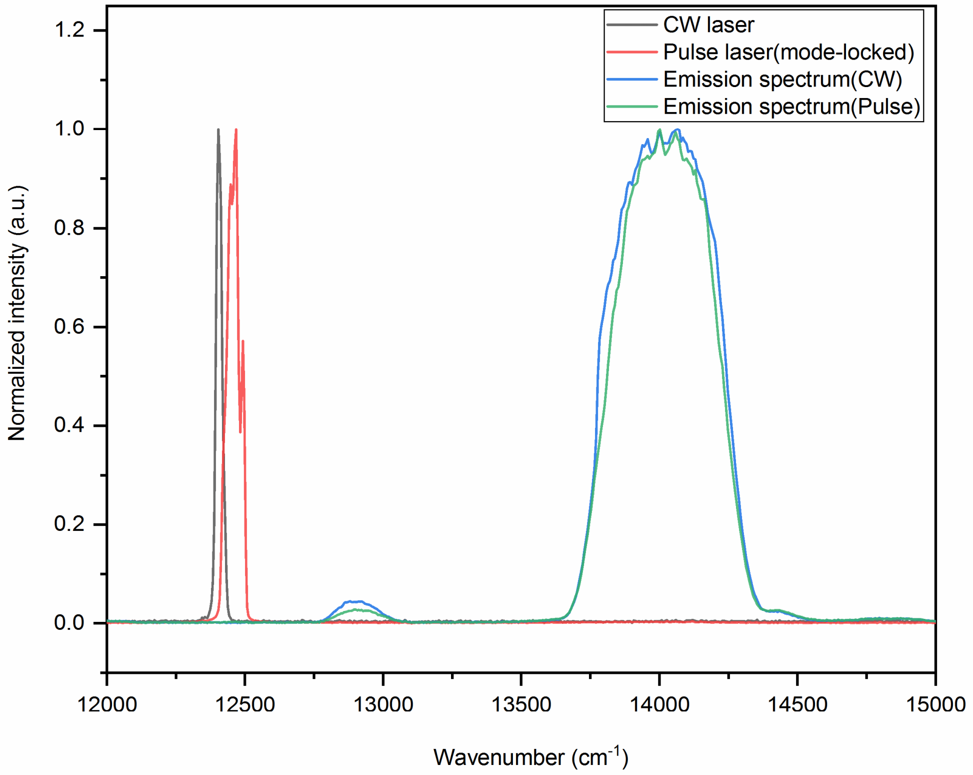

Fig. S6. BPAWR-SAM spectra obtained during the time-delay measurements. The emission wavelength of the CW laser before the mode-locked is 806.2 nm, and the wavelength is shifted to 802.0 nm after the mode-locked. The relative peak positions of the emission peaks at the two wavelengths did not change significantly, and the emission peaks corresponding to BPAWR-SACM appeared at 12912.0 cm^{-1} and 14002.3 cm^{-1}.

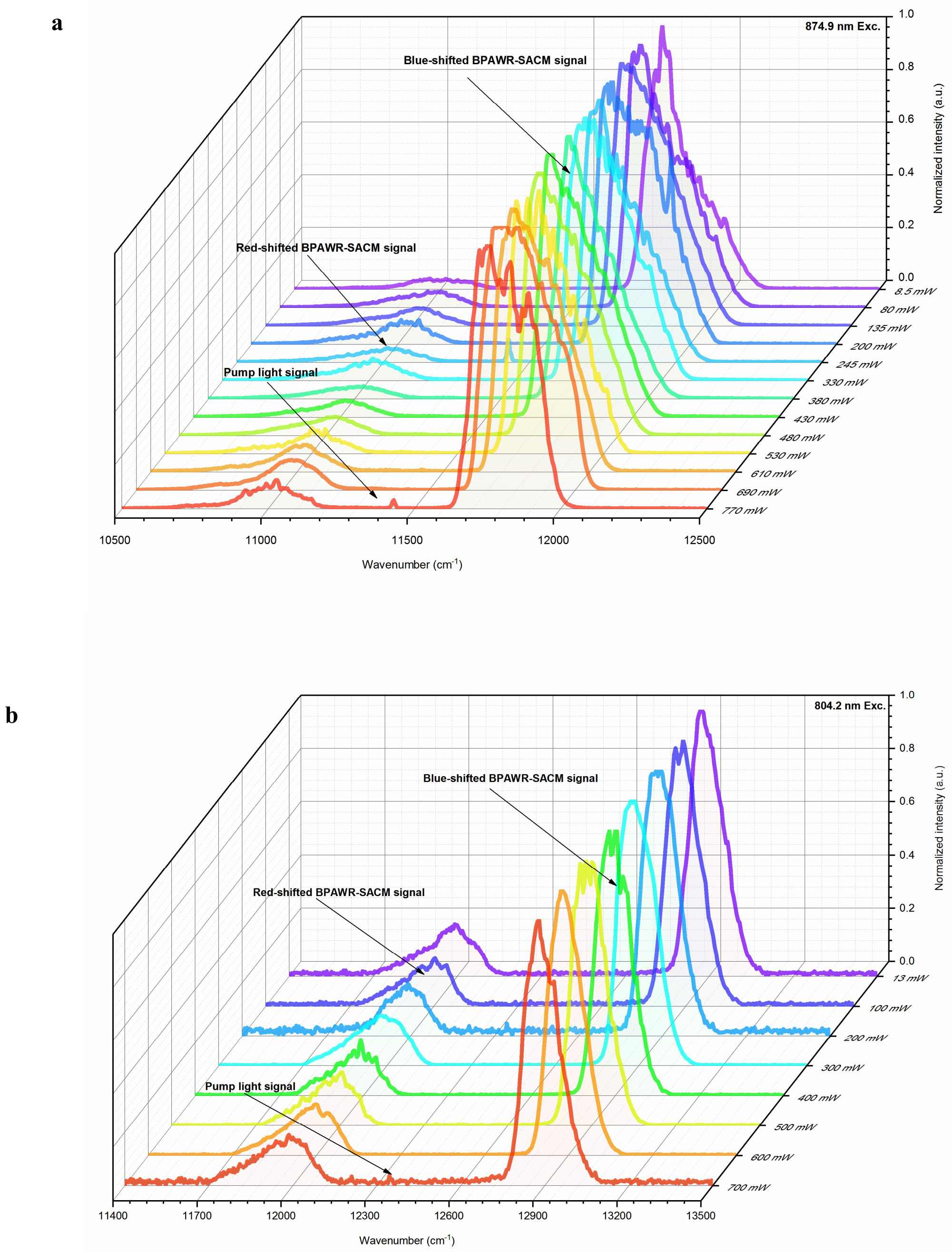

Fig. S7. Emission spectra obtained at different excitation powers of 874.9 nm laser (a) and 804.2 nm laser (b). The corresponding BPAWR-SACM emission peak signal can be obtained with the laser power of 8.5 mW and 13 mW, respectively.

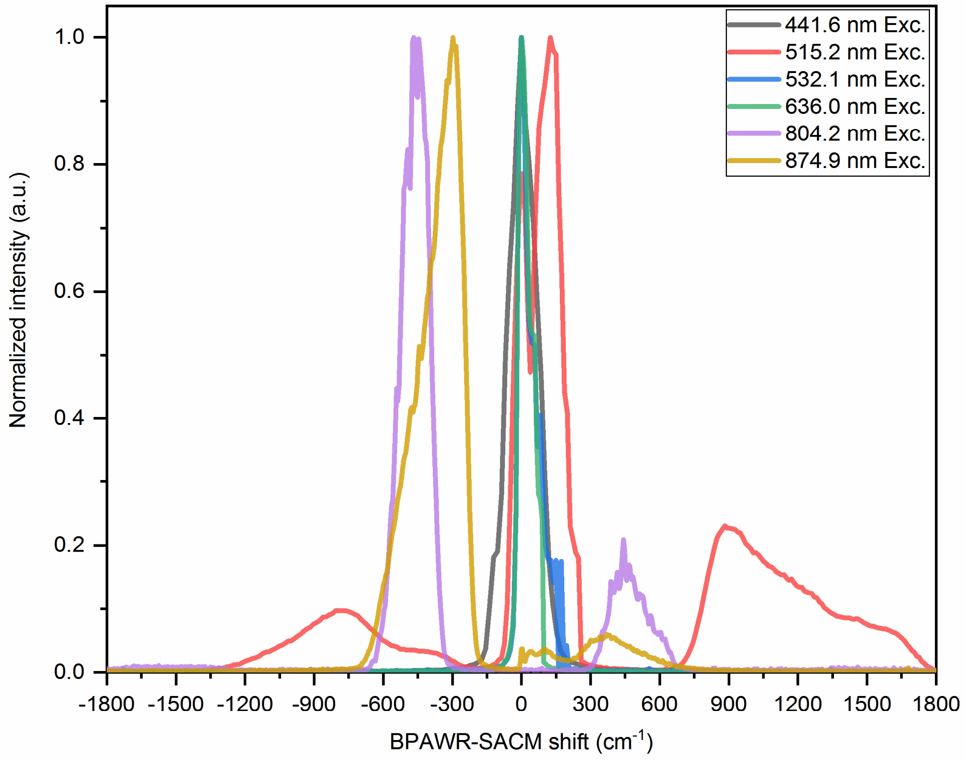

Fig. S8. Normalized BPAWR-SACM shift spectra at different excitation wavelengths. Only when the wavelength of the excitation light corresponds to the energy difference required for the energy level transition of Nd^{3+} ions, a significant BPAWR-SACM signal can be generated. The red-shifted and blue-shifted signals are asymmetric, and the peak position shifts of the corresponding emission spectra at different excitation wavelengths are not consistent.

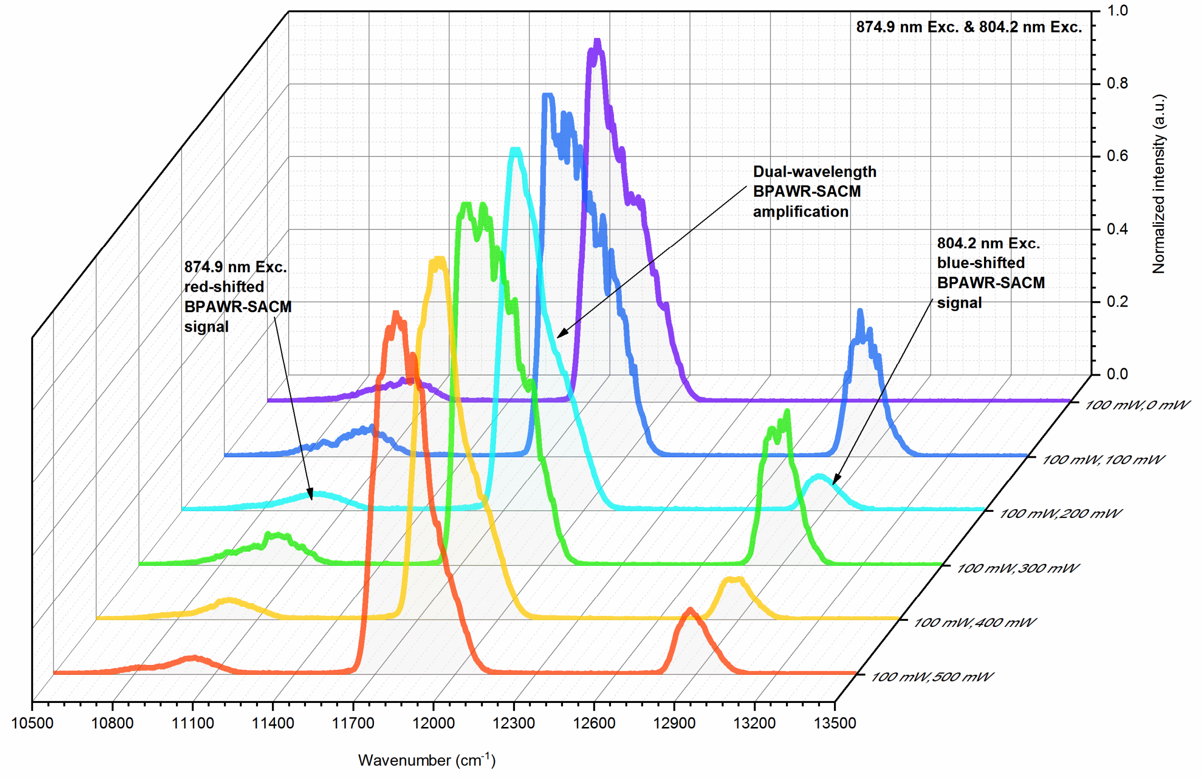

Fig. S9. Signal amplification by the dual-wavelength pump. Normalized emission spectra obtained by maintaining the laser's power at 874.9 nm constant and changing the power at 804.2 nm alone.

Table S1. Blue-shifted and red-shifted wavenumber of dual-wavelength pumps with varying powers. The red-shifted wavenumber corresponds to the excitation of 874.9 nm laser. And the blue-shifted wavenumber corresponds to the excitation of 804.2 nm laser.

Pump power (mW)		Shifted wavenumber (cm^{-1})	
874.9 nm Exc.	804.2 nm Exc.	Red-shifted	Blue-shifted
0	100	/	457.7
100	100	271.2	761.7
200	100	332.4	682.6
300	100	342.1	660.5
400	100	323.6	709.0
500	100	328.0	704.6
100	100	271.2	761.7
200	200	345.6	687.0
300	300	328.0	709.0
